# Editor's Letter-Box

**Published:** 1909-10-02

**Authors:** T. X. Murphy

**Affiliations:** 16 Pembridge Crescent, W.


### Editor's Letter-Box.

[Our Correspondents are reminded that prolixity is a great bar to publication, and that brevity of style and conciseness of statement greatly facilitate early insertion.]

Dear Sir,—I am directed to call your attention to a review on "Sprains and Allied Injuries of Joints" which appeared in your issue of September 18. While we were gratified by the appreciation of the reviewer, there appears a very careless mistake in the title of the book which is calculated to damage the work in question and which is extremely annoying to the author. If you will refer to the title of the book you will see that the author's qualifications are M.D. and M.C. Edinburgh, also that he is Honorary Surgeon to the Radcliffe Infirmary and Lichfield Lecturer in Surgery to the University of Oxford. Further, that the book is published by us and that its price is 7s. 6d. net, whereas in your review the author appears as Lichfield Assistant Pathologist, Edinburgh Royal Infirmary, and the book is said to be published by John Bale, Sons and Danielsson, at 2s. 6d. net. My directors trust that you will correct this mistake in your next issue, giving it as much prominence as possible. Very truly yours,

T. X. Murphy, Med. Ed.

16 Pembridge Crescent, W., September 23.

[We regret that owing to a printer's error the mistakes occurred in the paragraph dealing with the book in question. The price and publisher have already been given correctly in last week's issue.—Ed. The Hospital.]

